# West Nile Virus in Common Wild Avian Species in Israel

**DOI:** 10.3390/pathogens11010107

**Published:** 2022-01-17

**Authors:** Gili Schvartz, Sharon Tirosh-Levy, Shahar Bider, Avishai Lublin, Yigal Farnoushi, Oran Erster, Amir Steinman

**Affiliations:** 1 Koret School of Veterinary Medicine, The Robert H. Smith Faculty of Agriculture, Food and Environment, The Hebrew University of Jerusalem, Rehovot 7610001, Israel; giliun@gmail.com (G.S.); sharon.tirosh@mail.huji.ac.il (S.T.-L.); shahar.bider@mail.huji.ac.il (S.B.); 2 Department of Virology, Kimron Veterinary Institute, Beit Dagan 5025001, Israel; Oran.Erster@sheba.health.gov.il; 3 Department of Avian Diseases, Kimron Veterinary Institute, Beit Dagan 5025001, Israel; avishailublin@yahoo.com (A.L.); pop5552003@yahoo.com (Y.F.)

**Keywords:** West Nile virus, Israel, wild birds, 2018, qPCR

## Abstract

In order to evaluate the contribution of different wild bird species to West Nile virus (WNV) circulation in Israel, during the months preceding the 2018 outbreak that occurred in Israel, we randomly sampled 136 frozen carcasses of a variety of avian species. Visceral and central nervous system (CNS) tissue pools were tested using WNV NS2A RT qPCR assay; of those, 15 (11.03%, 95% CI: 6.31–17.54%) tissue pools were positive. A total of 13 out of 15 WNV RT qPCR positive samples were successfully sequenced. Phylogenetic analysis indicated that all WNV isolates were identified as lineage 1 and all categorized as cluster 2 eastern European. Our results indicated that WNV isolates that circulated within the surveyed wild birds in spring 2018 were closely related to several of the isolates of the previously reported 2018 outbreak in birds in Israel and that the majority of infected birds were of local species.

## 1. Introduction

West Nile virus (WNV) is an enveloped RNA virus of the *Flaviviridae* family. It is transmitted mainly to avian species, but also to some mammalian hosts, via arthropod vectors, specifically ornithophilic mosquito species [1]. WNV was first described in Uganda in 1937, where it was isolated from the blood of a native woman as a cause of a febrile disease [2]. Since then, sporadic cases and several outbreaks were reported in Africa, the Middle East, Europe, and Asia [3]. In 1999, the virus was introduced to the United States and was characterized by significant avian morbidity (mainly *Corvidae* spp.) in the New York area, following by morbidity in humans. Over a decade, WNV spread to the western US, Canada, and central America, and is now recognized in all continents except Antarctica [4]. 

The host range for WNV is wide and includes over 300 species of birds [5,6], mammals, reptiles, and amphibians [7,8]. Avian species, especially wild birds, are considered the main reservoir of WNV that is circulating in an enzootic cycle between birds and mosquitoes [8]. Host sensitivity to WNV infection, as well as the competence to inhabit virus replication adequate for infecting a new vector, varies between species [5,8,9,10]. The replication of WNV in birds is significantly higher compared to other species. Some bird species may have high viremia for extended time without clinical signs, therefore could contribute to the geographical extension of the virus during annual migrations. However, several other bird species are highly sensitive to infection and will show significant clinical signs that range between weakness, ataxia, circling, and even death [5,11,12,13]. Clinical infection in incidental, dead-end hosts, such as humans and horses, is usually asymptomatic, but may also result in mild febrile disease with flu-like symptoms, and occasionally will result in neuroinvasive disease, manifested with neurological deficits (ataxia, weakness, mental disturbances) and possibly death [13,14,15,16]. 

Experimental inoculation studies in different bird species indicated a variety of virus replication sites, sensitivities for infection, and length and level of viremia [7,11,12]. Local avian species’ sensitivity may vary geographically, e.g., between different continents, possibly due to WNV genotype variation and/or previous cross-immunity to close flaviviruses [10,11,17,18].

Israel, located on a central junction of migratory routes between Africa, Europe, and Asia, is a home for a variety of permanent, local-native, and a few invading avian species. It also serves as an important migratory station for rest and/or seasonal nesting. WNV has been endemic in Israel since 1952 [19]. Sporadic cases were reported in Israel until the late 1990s, where increased morbidity and mortality occurred in geese and wild birds, especially white storks [20,21]. In 2000, a major outbreak of WNV encephalitis was reported in hundreds of humans and dozens of horses in Israel [16,20,22]. Since then, minor outbreaks were reported sporadically, the most recent in 2018, where increased morbidity and mortality were observed in wild avian species concurrent with increased morbidity and mortality in humans [23,24]. Several cases were reported in horses, and four molecular isolations were identified [23]. All animal cases were phylogenetically identified as lineage 1, cluster 2 and 4. Interestingly, this type has not been reported in humans and mosquitoes, despite continuous surveillance, since 2004 [24,25,26]. Until 2018, all reported cases of WNV from animals in Israel were of lineage 1 as well [23,24]. Local circulation and the introduction of WNV in Israel is facilitated via viremic birds, and results in occasional infection of humans and horses [16,20,23,24]. In order to evaluate the contribution of different wild bird species to WNV circulation in Israel, this study focused on the detection of WNV RNA in migrating and local birds admitted to the Israeli Wild Animal Veterinary Hospital (IWAVH).

## 2. Results

The randomly collected frozen carcasses included local and migrating avian species, mainly *Passeriformes*, but also *Anseriformes*, *Columbiformes*, *Accipitriformes*, and *Strigiformes*, as detailed in Table 1. The majority of the examined birds (60.6%) were of local species (Table 1), while only 11.7% were purely migrating birds, and the others were considered both migratory and resident in Israel. The birds originated from different parts of Israel; however, since the geographical data were only available for 29 birds, the distribution regarding the geographical origin was not analyzed. 

Visceral and central nervous system (CNS) tissue pools from 136 birds were tested with WNV NS2A RT qPCR assay; of those, 15 (11.03%, 95% CI: 6.31–17.54%) tissue pools were positive. Most of the positive birds were resident *Passeriformes* birds. The most common positive species was *Corvus cornix* (*n* = 6, Table 1), with half of the crows tested identified as being infected with WNV. None of the positive birds were of strictly migratory species: 12 were of local species (14.5% of all resident birds in this survey) and three of species that are both resident and migratory (7.9%). However, this difference was not statistically significant (*p* = 0.215).

**Figure 1:**
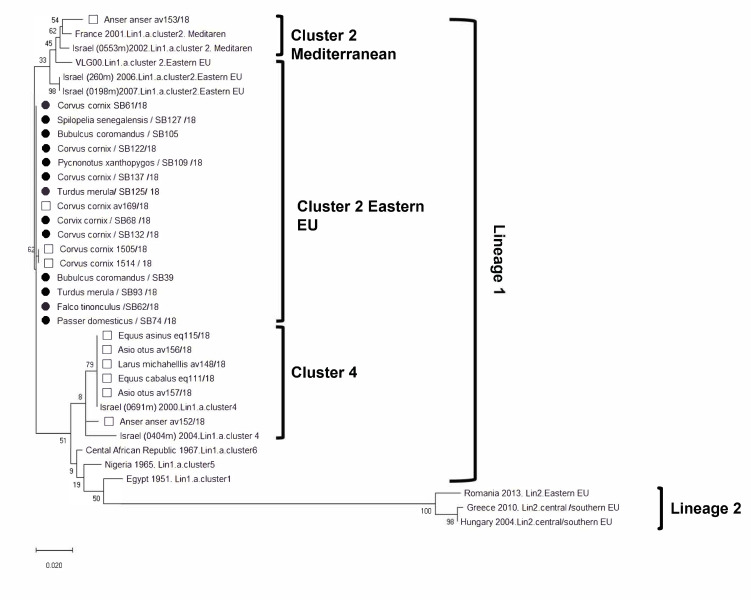
Phylogenetic analysis of WNV from avian hosts studied in Israel during 2018. The analysis was conducted on nucleotide sequences of the genes encoding the capsid, pre-membrane protein, and membrane protein, using the maximum likelihood method Tamura-Nei model, implemented in MEGA X-2 software. The robustness of branching pattern was tested by 1000 bootstrap replications. The rates among sites algorithm used was uniformly distributed. The bar denotes 0.02 nucleotide substitutions per site. Lineage1 and 2 reference strains are present with country and year of isolation. Sequences marked with black circles were retrospectively isolated from wild birds that died in spring 2018 in Israel. Sequences marked with white squares were isolated in Israel from wild birds and horses in summer 2018.

Thirteen out of fifteen WNV RT qPCR positive samples were successfully sequenced. Phylogenetic analysis indicated that all WNV isolates in this study (marked with black diamonds) (Figure 1) were identified as lineage 1 and all categorized as cluster 2 eastern European (Figure S1). As expected, the sequences obtained in this study were closely related to the isolates of the previously reported 2018 outbreak in birds in Israel [23]. 

## 3. Discussion

The epidemiologic and phylogenetic analysis of the 2018 WNV outbreak in Israel that resulted in 139 clinical cases in humans, including seven fatalities, indicated that only lineage 1 cluster 2 WNV circulated in humans and mosquitoes that year [24]. The phylogenetic tree that was conducted in that study demonstrated that all five samples obtained from humans clustered with the eastern European subtype of WNV lineage 1, cluster 2. The WNV strains found in mosquitoes belonged to both the Mediterranean and eastern European subtypes of WNV lineage 1, cluster 2 [24]. This is in contrast to the phylogenetic analysis of the clinical cases in equids and birds from summer 2018, where both cluster 2 and 4 were identified [23]. In detail, samples from one horse, one donkey, and two birds were grouped together on cluster 4. Samples from one horse and one bird were on cluster 2 Mediterranean, and seven samples from birds were on cluster 2 eastern European [23]. The phylogenetic analysis in the current study indicate that all WNV isolates that were collected from the carcasses of birds during the months May, June, and the beginning of July 2018 were identified as lineage 1, and all classified as cluster 2 eastern European. The sequences obtained in this study were closely related to several of the isolates of the previously reported 2018 outbreak in birds in Israel. The combined data from the 2018 human and animal WNV cases suggested that there was no common virulent WNV strain to the 2018 European outbreaks (mainly associated with lineage 2), and the 2018 outbreak in Israel [23,24]. 

While our sampled population was limited in size and randomly selected, it was nevertheless characterized by a relatively high variety of local and migrating species. Approximately 60% of birds’ species were residents in Israel, 11.7% were strictly migrating, and the rest were both migrating and residing birds. WNV-infected birds were mostly of local species. Three positive birds were both local and migrating. Although it cannot be definitively concluded, we assume that the increased morbidity during 2018 was mainly a result of increased local viral activity and transmission within the vectors and local reservoir birds (and onto dead-end hosts), and not necessarily introduced into Israel from an external source, e.g., migrating birds [27,28,29,30]. 

The majority of the birds examined in this study were *Passeriformes* birds, which include many species that serve as major reservoirs and are very sensitive to WNV infection [5,7,10,11,31,32]. Consistent with our results, other studies reported varied populations of bird species that are sensitive to and are involved in the WNV transmission cycle, especially local species, which are abundant and located in the vicinity of dense human population and vector foci [1,31,32,33]. Besides *Passeriformes*, the significant exposure and sensitivity of *Columbiformes* to WNV have been previously reported [34,35]. Our results also indicated viremic *Columbiformes* species that need to be considered as a potentially significant reservoir in the local transmission cycle of WNV in Israel.

The transmission of WNV in Israel during 2018 started and peaked earlier than the previously reported outbreak in 2015 [26]. Human and animal clinical cases usually peak in August to September [36]. Israeli public meteorological data (https://www.israelweather.co.il/forecast/avgYearTemp.html accessed on 23 November 2021) indicated that the spring months of 2018, February, March, and May, were warmer (+3.4, +4.7, and +2.4 °C, respectively) than the average temperature (Israel’s average temperature 2009–2021). April was not significantly different between 2015 and 2018 (2015 was the last reported outbreak before 2018) [26]. A comprehensive comparison with archived local official diagnostic data from mosquitoes, humans, and animals, and meteorological data from previous decades, are needed to conclusively determine whether climate conditions were associated with the recent outbreaks in Israel. It is conceivable, nevertheless, that an elevated temperature is associated with increased local virus transmission due to increased vector activity, as was suggested previously [27,28,37]. 

Based on a limited population size, our study indicate that a variety of local wild avian species may serve as an important part of the WNV transmission cycle in Israel. Many species of *Passeriformes* birds are considered to be highly sensitive to WNV and other arthropod transmitted flaviviruses such as Usutu virus, and for the last decades, also the main reservoir for WNV in Europe and America [1,13,33]. In this study, most of the positive birds (80%) were *Passeriformes,* which is a large avian order including around half of the known avian species. However, WNV-positive *Columbiformes, Accipitriformes*, and *Pelecaniformes* were also identified. This retrospective descriptive study sets the basis for continuous monitoring of WNV circulation within animals in Israel and for the acquisition of genetic data that will help to better understand the ecological aspect of this virus infection cycle. 

## 4. Materials and Methods

The IWAVH provides medical care to approximately 3000 animals annually, of which around 2000 are avian species—local, migrating, and some urban *Passeriformes*. Birds that did not survive were collected and kept frozen for prospective use for educational purposes in Tel Aviv University’s wild animal exhibitions. In order to detect WNV activity in avian species in Israel, as an indication for WNV circulation that preceded the 2018 outbreak [23], we randomly sampled 136 frozen carcasses of a variety of avian species, size, and age that were hospitalized and died during May, June, and the beginning of July 2018.

The avian carcasses were thawed overnight at 4 °C, followed by the harvesting of visceral and brain tissues. The samples were kept at −80 °C until further processing. Upon thawing, the tissue samples were homogenized in phosphate buffered saline (PBS) (in a volume ratio of 1:7) and then centrifuged at 1100g for 10 min, at 4 °C. Following incubation on ice for 10 min, supernatant was harvested for RNA extraction using the Ribospin vRD II extraction kit (GeneAll Biotechnology, Seoul, Korea) according to the manufacturer’s instructions. RNA was extracted and analyzed in pools (pools included visceral and CNS tissue per bird).

The following primer sets were used for the detection of WNV in the examined samples: for the NS2A test: forward primer: 5′-GGGCCTTCTGGTCGTGTTC-3′, reverse primer: 5′-GATCTTGGCYGTCCACCTC-3′, probe: 5′-CCACCCAGGAGGTCCTTCGCAA-3′) [38]. For one-step reverse transcription qPCR, we used AgPath-ID RT-PCR mix (Applied Biosystems™, by Thermo Fischer Scientific Waltham, MA USA, cat. no. 4387424) with the BioRad CFX96 thermal cycler (BioRad, Hercules, CA, USA). The reaction mix contained the following components: a mutant MMLV RT 1 µL, AmpliTaq Gold^®^ polymerase, 2X RT-PCR buffer 12.5 µL, ROX™ dye, detection enhancer 1.5 µL, PCR water 5.7 µL, forward primer 1 µL, reverse primer 1 µL, probe 0.4 µL added to 5 µL RNA. The reaction setup was as follows: RT at 45 °C for 10 min, activation at 95 °C for 10 min, 40 cycles of: 95 °C for 15 s, 60 °C for 45 s and plate read. 

The “Kunjin” (“KUN”) amplified segment (“KUN 108–988”) that includes a region spanning positions 108 and 998 in sequence number HM152775 [39] was used for the phylogenetic analysis. Forward primer: 5′-CCGGGCTGTCAATATGCTAAAACG-3′, reverse primer: 5′-GCTCCGGACACTCCCTCCAGG-3′. The reaction mix included 12.5 µL One-Step ToughMix (2×), forward primer 1 µL, reverse primer 1 µL, Gel Track Loading Dye (50×) 0.5 µL, qScript XLT One-Step RT (25×) 1 µL, nuclease-free water 4 µL, added to 5 µL RNA. The reaction setup was as follows: cDNA synthesis 48 °C, 20 min; initial denaturation 94 °C, 3 min, 40 cycles; denaturation: 94 °C, 10 to 20 s; annealing: 55–65 °C, 20 to 60 s; extension: 68 °C to 72 °C, 1 min, as per manufacturer’s instructions (Quantabio, Beverly, MA, USA).

Of 15 positive samples, 13 were successfully amplified. Sequence assembly and multiple alignments were performed using programs embedded in the Geneious 9.1.8 package (Biomatters, Auckland, New Zealand; www.geneious.com/about/ accessed on 23 November 2021). Sequences alignment included 10 WNV sequences from birds and equids diagnosed in the 2018 outbreak (of cluster 2; three eastern European and one Mediterranean and six from cluster 4, Table 2) and 13 reference WNV sequences of lineages 1 and 2. The alignment (780 bp) file was used to generate the phylogenetic tree using the Maximum Likelihood method constructed with MEGA X64.

## 5. Conclusions

WNV is endemic in Israel and may occur at higher risk when the average annual temperature is increased. Our results suggest that WNV circulation within local birds may have a significant role in establishing and maintaining increased WNV activity. Further continuous surveillance by examining birds involved in the WNV transmission cycle is needed to establish a comprehensive database that will allow the identification of avian species in Israel that are at a high risk for WNV transmission, and/or are highly sensitive to WNV infection. 

## Figures and Tables

**Table 1:** West Nile virus infection in various avian species (% in each avian species) and living type (L = local, M = migrating, B = both, *n* = number).

Type (L/M/B)	Avian Species	*n*	WNV Positive
L	*Acridotheres tristis*	3	0
L	*Bubulcus coromandus*	12	2 (16.7%)
L	*Carduelis carduelis*	1	0
L	*Cinnyris*	1	0
L	*Columba domestica*	8	0
L	*Corvus cornix*	12	6 (50%)
L	*Dendrocopos syriacus*	3	0
L	*Garrulus glandarius*	7	1 (14.3%)
L	*Halcyon smyrnensis*	2	0
L	*Myiopsitta monachus*	2	0
L	*Parus major*	2	0
L	*Passer domesticus*	11	1 (9.1%)
L	*Prinia gracilis*	2	0
L	*Psittacula krameri*	4	0
L	*Pycnonotus xanthopygos*	8	1 (12.5%)
L	*Spilopelia senegalensis*	4	1 (25%)
B	*Anas platyrhynchos*	1	0
B	*Corvus monedula*	1	0
B	*Falco tinnunculus*	19	1 (5.3%)
B	*Hirundo rustica*	1	0
B	*Plegadis falcinellus*	1	0
B	*Turdus merula*	7	2 (28.6%)
B	*Upopa epops*	7	0
B	*Venallus spinosus*	1	0
M	*Apus apus*	7	0
M	*Asio otus*	1	0
M	*Buteo buteo*	1	0
M	*Clamator glandarius*	1	0
M	*Coturnix coturnix*	1	0
M	*Otus scops*	3	0
M	*Streptopelia turtur*	1	0
M	*Sylvia melanocephala*	1	0
Total	L: 60.6%, B: 27.7%, M: 11.7%	136	15 (11.03%)

**Table 2:** GenBank accession numbers.

GenBank Accession No.	KVI/Avian Diseases No.	Sequence:
2540881	327316	*Corvus cornix* av169/18
OL365014	1105/2	*Anser anser* av153/18
MK343718.1	1038	*Larus michahellis* 148/18
OL365013	1105/1	*Anser anser* av152/18
MK343719.1	1505	*Corvus cornix* 1505/18
OL450362	1514	*Corvus cornix* 1514/18
OL450363	1131	*Asio otus* 157/18
MT828576.1	324085	*Equus cabalus* 111/18
2540897	1111	*Asio otus* 156/18
MT828577.1	325209	*Equus asinus* 115/18

## Data Availability

Data are contained within the article or Supplementary Material.

## Supplementary Materials

The following are available online at https://www.mdpi.com/article/10.3390/pathogens11010107/s1, Figure S1: Sequence alignment for the phylogenetic analysis.
