# Supplementary material for: West Nile Virus in Common Wild Avian Species in Israel
Source: Pathogens. 2022 Jan 17;11(1):107. doi: 10.3390/pathogens11010107 (PMC8780237; doi:10.3390/pathogens11010107)
Supplement: Supplementary file 1 [file pathogens-1501343-supplementary.pdf › pathogens-1501343-supplementary.pdf]

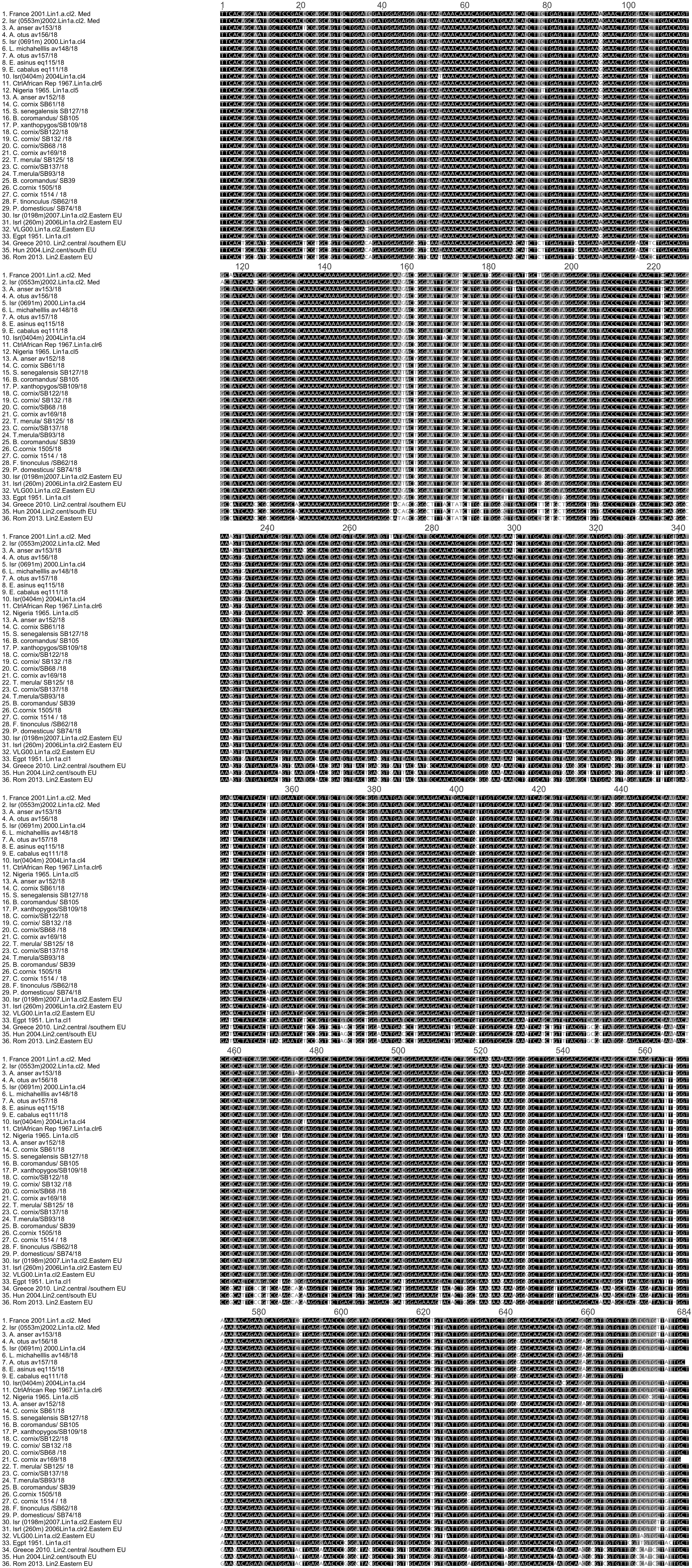
1. France 2001.Lin1.a.cl2. Med
2. Isr (0553m)2002.Lin1a.cl2. Med
3. A. anser av153/18
4. A. otus av156/18
5. Isr (0691m) 2000.Lin1a.cl4
6. L. michahellis av148/18
7. A. otus av157/18
8. E. asinus eq115/18
9. E. cabalus eq111/18
10. Isr(0404m) 2004Lin1a.cl4
11. CtrlAfrican Rep 1967.Lin1a.clr6
12. Nigeria 1965. Lin1a.cl5
13. A. anser av152/18
14. C. cornix SB61/18
15. S. senegalensis SB127/18
16. B. coromandus/ SB105
17. P. xanthopygos/SB109/18
18. C. cornix/SB122/18
19. C. cornix/ SB132 /18
20. C. cornix/SB68 /18
21. C. cornix av169/18
22. T. merula/ SB125/ 18
23. C. cornix/SB137/18
24. T.merula/SB93/18
25. B. coromandus/ SB39
26. C.cornix 1505/18
27. C. cornix 1514 / 18
28. F. tinonculus /SB62/18
29. P. domesticus/ SB74/18
30. Isr (0198m)2007.Lin1a.cl2.Eastern EU
31. Isrl (260m) 2006Lin1a.clr2.Eastern EU
32. VLG00.Lin1a.cl2.Eastern EU
33. Egpt 1951. Lin1a.cl1
34. Greece 2010. Lin2.central /southern EU
35. Hun 2004.Lin2.cent/south EU
36. Rom 2013. Lin2.Eastern EU
1 20 40 60 80 100
120 140 160 180 200 220
240 260 280 300 320 340
360 380 400 420 440
460 480 500 520 540 560
580 600 620 640 660 684
